# Insights into National Laboratory Newborn Screening and Future Prospects

**DOI:** 10.3390/medicina58020272

**Published:** 2022-02-11

**Authors:** Ahmed H. Mujamammi

**Affiliations:** Clinical Biochemistry Unit, Department of Pathology, College of Medicine, King Saud University, Riyadh 11461, Saudi Arabia; amujamammi@ksu.edu.sa

**Keywords:** newborn screening, national laboratory, inborn errors of metabolism, NGS, molecular diagnosis

## Abstract

Newborn screening (NBS) is a group of tests that check all newborns for certain rare conditions, covering several genetic or metabolic disorders. The laboratory NBS is performed through blood testing. However, the conditions that newborn babies are screened for vary from one country to another. Since NBS began in the 1960s, technological advances have enabled its expansion to include an increasing number of disorders, and there is a national trend to further expand the NBS program. The use of mass spectrometry (MS) for the diagnosis of inborn errors of metabolism (IEM) obviously helps in the expansion of the screening panels. This technology allows the detection of different metabolic disorders at one run, replacing the use of traditional techniques. Analysis of the targeted pathogenic gene variant is a routine application in the molecular techniques for the NBS program as a confirmatory testing to the positive laboratory screening results. Recently, a lot of molecular investigations, such as next generation sequencing (NGS), have been introduced in the routine NBS program. Nowadays, NGS techniques are widely used in the diagnosis of IMD where its results are rapid, confirmed and reliable, but, due to its uncertainties and the nature of IEM, it necessitates a holistic approach for diagnosis. However, various characteristics found in NGS make it a potentially powerful tool for NBS. A range of disorders can be analyzed with a single assay directly, and samples can reduce costs and can largely be automated. For the implementation of a robust technology such as NGS in a mass NBS program, the main focus should not be just technologically biased; it should also be tested for its long- and short-term impact on the family and the child. The crucial question here is whether large-scale genomic sequencing can provide useful medical information beyond what current NBS is already providing and at what economical and emotional cost? Currently, the topic of newborn genome sequencing as a public health initiative remains argumentative. Thus, this article seeks the answer to the question: NGS for newborn screening- are we there yet?

## 1. Introduction

Newborn screening (NBS) is a group of tests that check all newborns for certain rare conditions. This program covers several genetic or metabolic disorders, physical or functional, that affect the lives of newborns in one way or another. Most of these disorders may be without clear symptoms, but, in the long term, they may affect the infant’s life if left undiagnosed and untreated. Therefore, the goal of the early detection program is to identify these disorders in their infancy and programs for management and control for them are prepared to save the lives of those newborns.

NBS includes heel prick and urine samples for laboratory testing, a hearing screen and pulse oximetry. However, the conditions that newborn babies are screened for vary from one country to another.

The metabolic disorders are broadly classified into three parts: amino acid disorders, organic acid disorders and fatty acid disorders. In addition, various conditions are listed within each part. Amino acid disorders are caused by one of three events: enzymes that aid in the breakdown of specific amino acids fail to operate, enzymes that assist the body in excreting nitrogen contained in amino acids are unable to do so, or cofactors essential for these enzymes are not functioning. Subsequently, toxic levels of these precursors increase in the body, causing illnesses of varying degrees. Maple Syrup Urine Disease (MSUD) and Phenylketonuria (PKU) are two examples of aminoacidopathies [1].

Fatty acid disorders are caused by the malfunction of enzymes that help in converting fat into energy. When the body runs out of glucose (primary source of energy), fat must be used to supplement the creation of energy. The cells of the body suffer from an energy crisis when they do not have any glucose to convert and cannot use fat, which can lead to coma or death. Two examples of this type of disorder are medium-chain acyl-CoA dehydrogenase (MCAD) deficiency and very-long-chain acyl-CoA dehydrogenase (VLCAD) deficiency.

The third part is disorders of organic acid metabolism, characterized by the inability of an enzyme to break down amino acid, lipid or sugar intermediates. Toxic acids build up in the body when these compounds are not broken down, and, if not treated properly, these problems can lead to coma or death in the first month of a newborn’s life. Two examples of this category are glutaric academia type I (GA-I) and isovaleric academia (IVA).

## 2. The Historic Beginning of Laboratory NBS

The challenges raised by laboratory NBS and future technological advances are highlighted as it is attempted to maintain the success of NBS in the 21st century.

Laboratory NBS began as a blood test administered shortly after birth to prevent intellectual disability caused by untreated PKU, a rare, hereditary metabolic disorder in which affected individuals are unable to metabolize the amino acid phenylalanine, resulting in the accumulation of toxic metabolites and a lack of its essential product, tyrosine. For the past 60 years, NBS has served as a model for successful public health screening programs. However, the history of laboratory NBS is not without debate. With the introduction of tandem mass spectrometry (TMS), a method that has substantially increased the capability of detecting probable illnesses in asymptomatic newborns [2], several of these have changed [2]. NBS represents a success of the 20th century public health system. In the United States, four million infants are examined each year for a variety of diseases, the most frequent of which are congenital hypothyroidism (CH) and phenylketonuria (PKU) [3]. Almost all of the four million babies born each year are subjected to NBS, and around 12,500 babies are diagnosed with NBS issues each year [4]. No technique could successfully identify asymptomatic newborns with PKU within the first week of his/her life before Robert Guthrie [5] developed the bacterial inhibition assay (BIA) for PKU in 1961 [6]. Guthrie’s test, on the other hand, was inexpensive, simple and reliable. Any test utilized in a mass screening program had to have these properties. Dr. Robert Guthrie developed a BIA in 1960 to detect high blood phenylalanine levels before symptoms appeared. This finding allowed for the identification of infected individuals prior to the onset of symptoms, allowing them to be treated, and therefore avoiding the disease’s deadly effects [5].

## 3. Organizational Guidelines for NBS

In its early stages, the NBS program began by taking a blood sample from the newborn immediately after birth by sipping the heel of the foot, taking the blood sample on a blotting paper, then saving it and sending it to the laboratory for genetic testing to diagnose some genetic or metabolic disorders. This method was easy, accessible for all and at low cost, through which many life-threatening diseases were diagnosed for infants in the first weeks after birth, such as metabolic acidosis or neuromuscular disorders, and then programs for their control and treatment were developed. However, the presence of some biochemical substances in the solutions used in collecting and preserving diagnostic samples, such as heparin, and the circumstances surrounding its collection, such as blood transfusion for the newborn, all affect the values of the results of the examinations that are conducted for newborns, especially those under intensive care, so controls must be established to ensure that these factors do not affect the test results as much as possible [7]. Furthermore, the cards for the collection of the blood drops could contain a part for the anamnestic data collection, which could help the screening laboratory to correctly interpret the test results.

The development of such practice recommendations for use in NICUs is especially significant because many of the severe sequelae of metabolic illnesses can be avoided if recognized early. Furthermore, no standardized practice guidelines currently exist for determining who, whether primary care provider or birth hospital, is responsible for notifying the parent of a positive result and therefore ensuring invaluable follow-up care. Such standardized guidelines for screening practice are needed to prevent distressing neurologic sequelae for newborns or infants whose condition may otherwise be left unaddressed. The National Board of Surgery guidelines, produced at Johns Hopkins Hospital and Memorial Regional Hospital, is a good material to start with [8].

Scientific and technological advances have led to a significant expansion in the number of tests from an average of one to more than 50, and there is a national trend to further expand the NBS program. Many ethical, social and legal complications have emerged as a result of the rapid updates in the NBS programs that affected healthcare programs for the management of the detected cases [9]. In the United Kingdom, the NBS program is implemented based on policies and rules that are periodically reviewed at the national level, and the procedures for the program, from deleting, adding or modifying any examinations of the program, are not performed without careful review and approval of the relevant official authorities. In the United States, each state has developed its own NBS program that is aligned with the regulations of the federal advisory committee of inheritable diseases. Knowledge of the advances in NBS in different countries that have diverse types of healthcare systems would help in informing nurses about the totality of healthcare for newborns and assist them in becoming more knowledgeable about how international standards differ from those in the United States [10].

With a few notable exceptions, NBS programs participate in quality control programs much like any other laboratory. The cards used to collect blood spots account for a large part of the success of NBS programs. Preliminary studies using Robert Guthrie’s test for PKU stated elevated false positive rates that were attributed to a poorly selected type of card. This source of variation has been eliminated in most NBS programs through standardization of the approved sources of cards to be used for NBS. In most regions, an identification card is made for the newborn subjected to the NBS program containing all personal data such as name, age, gender and family information, and attached with it the nominated filter paper for its blood sample taken from the heel and the date of collection of the sample in order to distinguish the samples sent to the examining laboratory [11].

## 4. Laboratory NBS Worldwide

TMS for inborn errors of metabolism (IEM) diagnosis offers the potential to expand the NBS panels to encompass a large number of illnesses. This method allows diagnosis of many metabolic abnormalities in the same dry blood spot (DBS) in a single analytical run. TMS screening technology has quickly replaced the use of traditional techniques for the screening of genetic or metabolic disorders, which includes identification of one metabolite for each analysis for one disease at a time. Therefore, the use of TMS technology in the NBS examination provides a reliable measure of specificity and sensitivity in examination results where we can use only one metabolite for the identification of multiple disorders at the same time [12].

The use of TMS as a second level test has also made it possible to separate and quantify the isobaric amino acids leucine, isoleucine, valine and allo-isoleucine, increasing the specificity of the NBS result for urine with a maple syrup smell [13]. Another example in which this approach has been applied is in the detection of tyrosinemia type I. The NBS program of the North American state of Minnesota has implemented a TMS method to determine the concentration of succinyl acetone when the concentrations of tyrosine in the initial screening by the traditional method are above the established cut-off point. Recent studies report TMS has been used in screening for more than 50 IEMs using a dried blood spot in the neonatal period [14].

Implementing the NBS program is subjected to local resources and healthcare infrastructure [15]. Worldwide, there are extensive variations in the number of screened disorders ranging from one in some countries to 50 in other countries, Table 1 illustrates some. In China, for example, the NBS began in 1981 with only two disorders, CH and PKU, before being expanded [16]. The NBS for CH in Canada began as a pilot initiative in 1974 and was expanded to include all neonates [17,18]. Furthermore, diverse techniques and screening for different illnesses are used by the NBS Programs in Western Europe, North America and Australia [16,17]. CH is one of the top causes of mental retardation and it is completely preventable [18]. It can either be permanent in the form of dysgenesis, agenesis or dyshormonogenesis, or it can be transient [17,18]. In Saudi Arabia, the NBS program started in 2005 through collaboration between some health-providing agencies in the Kingdom including; the Ministry of Health (MOH), King Faisal Specialist Hospital & Research Center (KFSHRC) and King Salman Center for Disability Research (KSCDR) to screen for 17 disorders, and in January 2019, three more disorders were added to the national screening program [15].

## 5. Advances in Screening Methodology

NBS has grown slowly but steadily in the United States during the last four decades. Not simply the medical needs and benefits of the screened neonates, but also the interests of the family and society at large are now deemed crucial to its ethical validity [19]. With the expansion of its moral focus, NBS is now used to detect even untreatable diseases and reveal genetic tendencies for late-onset genetic disorders. Geneticists aim to create effective treatments for some of the worst genetic disorders by including these conditions in screening programs, but, for the most part, such treatments will take many years to develop. NBS continues to be a model population genetic screening program [20], based on a progressive and successful history. Within the last five years, most screening programs in the USA have begun to use TMS as the principal tool for analyzing blood spots [21]. This technology has reduced false positive findings, though they still exist, and it has simplified the screening process: the test can detect over 40 metabolic disorders [3].

Before the use of TMS in the NBS program, there was a need to perform microbial assay testing for each case. Currently, these separate assays are required only for a few cases of either endocrine disorders (i.e., CH and CAH), hemoglobinopathies and thalassemias (i.e., sickle cell disease, S-β thalassemia), or other cases (e.g., biotinidase, cystic fibrosis and hearing disorders). A mass spectrometer is an instrument that separates and quantitates ions based on their mass to charge (*m*/*z*) ratio. In fact, the extracted molecules of each sample are delivered to the ion source of the mass spectrometer (by a liquid chromatography system) that generates a fine spray of charged droplets from which ions are emitted as the solvent evaporates. These ions are introduced into the mass analyzer for detection.

In the MS–MS system, the ions beam is focused and introduced into the first mass filtering quadrupole, Q1, where the ions are separated by their *m*/*z* ratio, and then they are moved along into the collision cell. Inside the collision cell the ions collide with inert gas molecules and fragment into smaller ions. The product ions are then introduced into a quadrupole, Q3, where they are separated by their *m*/*z* values and finally sent to the detector [22]. https://bioethicsarchive.georgetown.edu/pcbe/background/newborn_screening_crowe.html (accessed on 5 September 2021)—endnote12.

The metabolism within the mitochondria produces many metabolites that help to diagnose many genetic disorders, especially those affecting fatty acid oxidation in IDMs. These biomarkers include L-carnitine and acylcarnitines, which are not routinely measured in these tests despite their clinical importance outside of these disorders. Measurements of the carnitine pool have been used to identify the disease and predict mortality among disorders, such as diabetes, sepsis, cancer and heart failure, as well as identify subjects facing adverse drug reactions from different medications such as cisplatin, clofazimine, cyclosporine, propofol, valproic acid and zidovudine [23].

The quantification of acylcarnitines and amino acids is important in detecting disorders. High or low levels of these indicate that the neonate has a potentially dangerous metabolic disorder. In such cases, there is impaired function of the enzymes responsible for the breakdown of amino acids or the conversion of fat into energy. Consequently, the metabolites accumulate to toxic quantities. The determination of the accumulated compounds in the blood of newborns can be analyzed by TMS, the most available reliable method widely used in many regions [24]. https://bioethicsarchive.georgetown.edu/pcbe/background/newborn_screening_crowe.html (accessed on 5 September 2021)—endnote13.

Jointly, the NBS aims for the early detection and treatment of newborns with certain conditions before the symptoms’ onset for the reduction of morbidity and mortality. Furthermore, there are criteria for identifying the disorders for screening known as the Wilson and Jungner Criteria for Disease Screening [25].

### Wilson and Jungner Criteria for Disease Screening

1. The condition sought should be an important health problem.
2. There should be an accepted treatment for patients with recognized disease.
3. Facilities for diagnosis and treatment should be available.
4. There should be a recognizable latent or early symptomatic stage.
5. There should be a suitable test or examination.
6. The test should be acceptable to the population.
7. The natural history of the condition, including development from latent to declared disease, should be adequately understood.
8. There should be an agreed policy on whom to treat as patients.
9. The cost of case-finding (including diagnosis and treatment of patients diagnosed) should be economically balanced in relation to possible expenditure on medical care as a whole.
10. Case-finding should be a continuing process and not a “once and for all” project.

Reproduced from https://www.scielosp.org/article/bwho/2008.v86n4/317-319/ (accessed on 5 September 2021).

New technologies, including genomic sequencing, have increased the number of conditions that can be detected during the newborn period. The question of whether or not infants should be screened for conditions for which there is no treatment is debatable [26]. The prospect of conducting genomic sequencing as a part of NBS further complicates this debate. In 2013, the USA National Institutes of Health (NIH) provided grants totaling USD 25 million to four institutions to “Identify the opportunities for application of genomic sequencing for screening in the newborn period and the possible problems and implications associated with applying this test” [27]. These grants aimed to encourage researchers to increase the available data scale for analysis in the newborn period and to expand the understanding of specific diseases detectable by NBS through DNA-based analysis. Despite the enthusiasm for this technology, the state NBS programs and the United States’ healthcare delivery system lack the personnel necessary to interpret sequence variants and provide appropriate clinical follow-up to the infants who would be identified as having an increased risk of disease on such a large scale [28].

TMS’s growth in NBS is already putting strain on the current NBS infrastructure. Meanwhile, DNA microarrays, the next technological advance, is on the horizon and fast approaching [29]. Through NBS, it will be possible to test for hundreds of possibly disease-causing genetic variants [30,31]. The challenge of distinguishing disease-causing from benign mutations appears great as it should seek to avoid labeling newborns as potentially diseased and causing undue distress and “medicalization” [32].

Considerable resources are needed to refine the technology applied in expanding NBS in the community. Not only is the technology needed to initiate well-functioning programs for NBS, but also the services of optimum detection and follow-up of the spread of the disorders is required. According to Tarini 2007, “Many questions about the process of expanding newborn screening need a definitive answer. If it is proven that the examination of a newborn is positive for a certain disorder, this may represent a psychological burden on the doctor supervising the case. How to inform the family of the newborn with the positive result? And how he will determine the plan of management and follow-up for this case? What are the psychological and social burdens on the family of that newborn?” All these inquiries should be answered by coordination between families, specialists and primary care physicians to ensure the achievement of the NBS program in the 21st century [2].

## 6. National Laboratory NBS at Saudi Arabia

The Ministry of Health (MOH) in Saudi Arabia continues to activate the national NBS program to eliminate disabilities. This program is designed for the early detection of any genetic disorders causing severe complications across the Kingdom, including mental and motor disabilities, weak growth and premature death. Failure to recognize and treat such disorders early, according to the MOH, will result in indefinable consequences, as well as a significant social and mental load on the infant’s parents, which the program aims to alleviate. Furthermore, the program aims to screen all newborns in their first 24–72 h of life and is currently being implemented in 183 of the Kingdom’s 188 maternity and children’s hospitals in order to detect disorders early and provide needed healthcare as soon as possible in order to avoid long-term complications.

The national NBS program provides one general objective; namely, early screening of the newborn reduces morbidity and disability rates caused by genetic disorders (endocrine and metabolic diseases covered by the program). The program also has detailed objectives, including: extending the program’s coverage to include maternity hospitals, boosting the program’s efficiency at all levels, engaging the community, raising the community’s awareness about genetic disorders included in the program, continuing evaluation to improve the program and increase its performance, and generating a database of all national genetic diseases [33].

## 7. Reason for Limiting the Number of Screened National NBS Disorders

In a country such as Saudi Arabia, which is reported to have one of the highest prevalence of metabolic disorders in the world, applying the existing infrastructure for early diagnosis and treatment is achieved via establishing a national screening policy capable of encouraging Saudis to screen for these conditions as early as possible [34]. According to the Ministry of Health, 60% of Saudis are obese or overweight, with no proper physical activity, 75% do not have routine check-ups, and 18% of Saudis are smokers. Furthermore, in comparison to Western European populations, 46 percent of deaths from noncommunicable diseases occur in younger people. The number of Saudis with chronic diseases is expected to rise from 5 million to 10 million by 2030, according to the existing prevalence of health risk determinants [35].

In 2016, the government of Saudi Arabia launched the National Transformation Program to ensure the realization of the Kingdom’s Vision 2030. A total of 24 governmental institutions participated in the implementation of this program with the aim of achieving the 96 strategic goals of the Kingdom’s Vision 2030, and raising the health of the community is one of the crucial strategic objectives of the vision to prevent or at least reduce health risks [36]. The Saudi MOH is leading this strategic transformation and providing an integrated model for healthcare in the Kingdom, involving actions focusing on diseases’ prevention and strengthening the primary healthcare system [35].

As part of the efforts to screen newborns in the Kingdom of Saudi Arabia, some separate and independent examinations were conducted in many areas outside the framework of the Kingdom’s national program. Three studies were found where screening for congenital anomalies and IEM were performed for newborns between 1980 and 1999 [37,38,39]. The findings of these investigations suggested the establishment of a Saudi NBS program. An early literature review by Afifi and Abdul-Jabbar published two years after the establishment of the Saudi NBS Program assessed the needs and challenges of the program. In their review, they justified the need for an NBS program by the high birth rate of the Saudi population and the higher incidence of newborn disorders in comparison to the USA and Japan populations, where approximately 500 cases could be detected on a yearly basis [40].

A retrospective study performed by Alfadhel et al. stated the results of 775,000 national NBS in Saudi Arabia between 2005 and 2012, analyzed in 139 hospitals [41]. A total of 743 cases were detected, where the most frequently reported cases were CH, CAH and PA. The authors also showed that the incidence of IEM was one of the highest worldwide, which could be due to consanguineous marriages.

## 8. Prevalence and Geographical Distribution among Saudi Arabia

One of the studies conducted by the team of the NBS program of the MOH at Eastern province, investigated the incidence of the 17 inherited metabolic and endocrine disorders in the Eastern and Al-Jawf Provinces of Saudi Arabia as part of the Saudi National NBS Program. In addition, studies within the National NBS program were conducted in the Eastern and Al-Jawf Provinces with the collaboration of 20 governmental hospitals of MOH during the period from January 2013 to July 2017. The blood samples were obtained from newborns via a heel prick on filter paper that was sent for biochemical and immunoassay testing for this retrospective study (199,143 newborns resulted in 264 positive cases). These results represent an incidence of 1:754.3 live births, and were compared with other regional, national and international studies. The most frequently reported cases were VLCAD deficiency (62 cases out of 199,143, incidence 1:3211) followed by CH (59 out of 199,143, incidence 1:3375) and CAH (25 out of 199,143, incidence 1:7965), PKU (18 out of 199,143, incidence 1:11,063), and the least common was 3-Hydroxy-3-Methylglutaryl-CoA Lyase Deficiency(HMG) (1 out of 199,143; incidence 1:199,143), while βKT deficiency was not detected (0 out of 199,143, incidence 0). The incidence of these seventeen inherited metabolic and endocrine disorders in the Eastern and Al-Jawf Provinces is among the most in the world, attributable to high rates of consanguineous marriage. The Alsherarat tribe in Jawf Province has demonstrated a high incidence of VLCAD, while Al-Ahsa has the highest incidence among the Eastern and Jawf Provinces. Comparison with other national studies uncovered geographical differences in the incidence of certain inherited metabolic disorders [42].

Another study on NBS, in the central province at Prince Sultan Military Medical City (PSMMC) in Riyadh, included the 17 disorders listed in the national NBS program and revealed that 56,632 newborns were screened with a coverage rate of 100% between January 2012 to December 2017. Thirty-eight cases were confirmed. The incidence of CH was 1:3775. PA was the most common metabolic disorder with an incidence of 1:14,158. VLCAD deficiency and GA1 had an incidence of 1:18,877 each. PKU, BTD deficiency, MSUD and CIT had an incidence of 1:28,316 each. However, GALT and MCC deficiency had the lowest incidence of 1:56,632 [15].

A third study performed at King Faisal Specialist Hospital and Research Center (KFSHRC) in Riyadh showed 396 patients with confirmed CF with positive cystic fibrosis transmembrane conductance regulator (CFTR) variants from January 1992 to December 2017. The age of the detected patients with cystic fibrosis when diagnosed was 3.4 (±SD 5) years, and the age of them when followed up was 10.2 (6.9) years. It was proven that the consanguinity between the parents of those patients with cystic fibrosis was 85% (Table 2).

The samples for cystic fibrosis (CF) were referred from the different regions of the Kingdom. The total number of the referred samples was 396, from which the eastern and northern regions referred 144 (37%) and 79 (20%) samples, respectively, and showed the highest prevalence of CF with c.2988 + 1G > A (3120 + 1G > A) and c.1418delG (p.Gly473GlufsX54) variants, respectively, prevailing in most of the Saudi CF patients.

Consanguineous marriage was prominent in these two regions that needed the implementation of a premarital counselling program [48]. The high rates of familial intermarriages in the eastern and northern regions is an important factor that has led to the areas sustaining the high incidence of CFTR variants among the carriers. Due to the high rate of consanguineous marriages in these two regions, it is noted that there is a high incidence of the CF variants (85%) in these two regions compared to (50%) the overall incidence among the Kingdom. These high rates of the prevalence of CF variants in these regions is mainly due to the consanguineous marriages that need condensed familial screening and premarital counselling.

**Figure 1:**
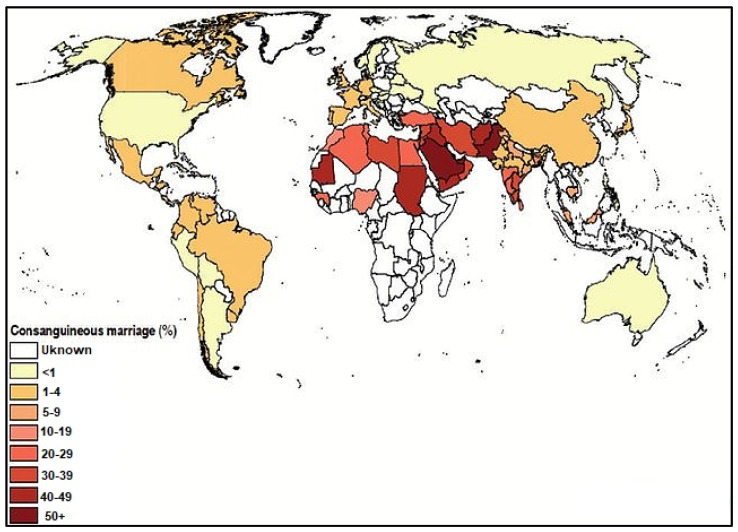
Global consanguinity map, updated October 2009 (Consang.net accessed on 5 September 2021) (Quoted with permission from the article titled: Towards a Uniform Newborn Screening Panel in the Kingdom of Saudi Arabia. Dr. Ahmed Bashir et al.).

In the following figure, there is a percentage distribution of consanguineous marriages across the globe (Figure 1).

## 9. Current Methodologies in Laboratory Testing

### Genetic Testing for NBS

The analysis of targeted pathogenic gene variants is a routine application in the molecular techniques for the NBS program, which is performed as a confirmative investigation after the biochemical or enzymatic testing has been performed and proven positive cases. Recently, a lot of molecular investigations based on DNA analysis and sequencing have been introduced in the routine NBS program.

Considering the experience of the Wisconsin program and what has been reported in the literature, the current practice regarding molecular technologies in routine NBS practice in the area of genomic and precision medicine is presented. A description of the three applications is shown in the following table (Table 3) [49].

The “just-in-time” information of the gene variant is used to assist the physicians interpreting the findings of the performed biochemical and enzymatic investigations within the NBS screening program, to decide the best and most suitable time to communicate with the families about the detected positive cases and to discuss with them the future management plan. This “just-in-time” information is a good means for differentiation between a disorder subtype. As an example of this in the Wisconsin NBS program, the primary marker in galactosemia is the galactose-1-phosphate uridylyl transferase (GALT) enzyme activity. The application of gene analysis is indicated in cases of reduced or absent GALT activity. DNA is extracted from the dried blood spots and amplified through PCR reaction using the Tetra-primer ARMS–PCR to amplify different variant alleles that can be distinguished by agarose gel electrophoresis [50].

For quick detection of genetic markers for high-risk disorders, we can use the “just-in-time” information as a crucial method for instant intervention. Maple syrup urine disease is a rare disease affecting 1 in 197.714 live births in the USA in general, based on the available data of the NBS program, which proved that the severe form of the disease can affect more than 1:400 births due to the presence of a founder variant of BCKDHA (c.1312T>A, p.Tyr438Asn) [51,52]. Table 4 summarizes some novel, common and founder mutations in the Saudi population. Moreover, a list of primers for amplifying and sequencing of some targeted mutations are shown in (Table 5).

## 10. NGS in Hemoglobinopathies

Hemoglobinopathies, including sickle cell anemia and α-/β-thalassemia, are the most common monogenic diseases worldwide [53]. Hemoglobin disorders are worldwide distributed, where about 340.000 children are born yearly with one of the hemoglobinopathies; developing countries have the majority of these cases with a ratio of 90% of the total affected births worldwide, which adds a heavy burden on the economy and healthcare status of those countries [54].

The presence of the sickle cell (HbS) gene in Saudi Arabia was first reported by Lehmann et al. in 1963 (*Nature 198*, pp. 492–493). Later, Hb S, α- and β-thalassemia, glucose-6-phosphate dehydrogenase (G6PD) deficiency and other enzymopathies were shown to have a variable prevalence among different provinces of Saudi Arabia. Nationally, recent studies have shown α-globin gene arrangement and β-globin gene polymorphism [55].

Al-Qurashie et al. reported that the eastern region of Saudi Arabia has the highest prevalence rate of sickle cell disease, followed by the southern region and the western–northern region, while the northern region has the lowest prevalence rate [56]. A study was performed by Nasserulla et al. in the Eastern Province demonstrated the prevalence of homozygous sickle cell disease reached 2.3% in Qatif and 1.08% in Al Hasa, and hemoglobin in Bartas reached 28% in Qatif and 16% in Al-Hasa. In Al-Hasa and Qatif, the frequency of the sickle cell gene was between 0.1109 and 0.1545%. The authors concluded that the NBS program in this area should be of routine practice due to the high rate of consanguineous [57].

Screening and diagnosis of hemoglobinopathies are facilitated with the use of the next generation sequencing (NGS) test, where the coding regions on the hemoglobin genes, including the four modifier genes, were designed and targeted [58]. Moreover, prevention programs based on carrier screening and prenatal diagnosis have resulted in a continuous decline in rates of thalassemia major at birth in the Mediterranean region [59].

NGS has been shown to allow rapid, multiplex and high-throughput detection of genetic variants [60]. NGS tests that are applied in whole genome or exome analysis can be used also in clinical research as a tool for the detection of genetic disorders [61]. In Mendelian recessive diseases, the tests performed on the level of the individual patient or carrier population are validated using the NGS and target capture, where they yielded high-quality genotype calls and acceptable false positive and false negative rates and cost-effectiveness [62,63].

NGS technology was used in a UK PhD research study to facilitate diagnostic procedures because of its ability to identify single nucleotide changes and large rearrangements in a single investigation. Examination using this technology included selecting DNA samples from people with thalassemia-causing mutations, fragmenting these samples and preparing them for DNA sequencing. Illumina platform was used for sequencing the two genomic regions that are affected by the variants causing thalassemia after enriching it for using in-solution bait capture. NextGene software (SoftGenetics) was used to sequence “reads” of the DNA fragments after aligning it to a reference sequence of the human genome. Changes in coverage between test and negative control samples identified dosage changing events. The presence of sequences that spanned the breakpoint region identified the breakpoints of structural variants. To improve the method, the analysis of samples with known, previously identified variants and testing them in samples with unknown novel variants was performed. Gap-PCR and sanger sequencing confirmed the findings and, in conclusion, NGS is considered as a novel technique that can improve the current diagnostic standards.

An examination of the features of the structural rearrangements identified revealed that multiple mutagenic mechanisms contribute to the range of variants that affect the alpha and beta globin gene loci [64].

## 11. NGS in Aminoacidopathies

In the past, the diagnosis of IEM was based on clinical findings confirmed by laboratory investigations. At that time, the trials for genetic diagnosis were costly and may have failed as there may have been several phenotypes of a gene caused by different gene mutations [65].

Nowadays, NGS techniques are widely used in the diagnosis of IMD, where its results are rapid, confirmed and reliable, but, due to its uncertainties and the nature of IEM, it necessitates a holistic approach for diagnosis. Currently, the widely-used NGS techniques concentrate only on the diagnosis of a definite group of genetic disorders and overlapping phenotypes, and the entrance of a clinical exome approach has facilitated the instant assessment of diverse phenotypes [66,67].

With respect to IEM, studies of hyperphenylalaninemia, phenylketonuria [44], cerebral creatine deficiency [68], glycogen storage diseases [69] and mitochondrial diseases [70] have generally yielded good results. Currently, customized NGS techniques are implemented in clinical studies where most analytic studies used whole exome sequencing (WES) [71,72]. The use of NGS in the genetic testing of Mendelian inherited diseases in children is greatly cost saving [73].

## 12. Is It Visible That Nucleic Acid Sequencing Will Replace the Current Available Methodologies for Newborn Testing?

Since NBS began in the 1960s, technological advances have enabled its expansion to include an increasing number of disorders. Advances now make it possible to sequence a newborn’s genome rapidly and economically. WGS and WES clinical application is increasing rapidly with many challenges. Its implantation in the NBS requires further investigations for possible clinical profits and for the unique ethical challenges it presents [74].

Until now, Sanger sequencing has been the gold standard method for DNA sequencing. Using this technology, a major expedition called the Human Genome project started in 1990 and lasted for 13 long years wherein USD 3 billion was expended to determine the whole human genome sequence. Challenges to be addressed before the implementation of NGS in NBS include data storage, real cost, ethical considerations, insurance disputes and medical malpractice. All these challenges are detailed in the responsibility for the maintenance and governance of a large amount of data generated, the costs for data analysis, family notification, follow-ups and confirmatory testing for each test, and for all tests, reporting uncertain results to the parents, the responsibility for the privacy of stored data and results until the maturation of the child, sharing results regarding serious, preventable genetic disorders with at-risk close relatives, will the test information make it difficult for an individual to obtain health insurance with a predisposition to a genetic disease, and whether clinicians are properly trained to interpret data generated by genome sequencing [75].

If genomic sequencing were integrated into NBS, state NBS programs would have to decide which effects would be returned to infants’ families. In 2013, the American College of Medical Genetics and Genomics (ACMG) [76] published a listing of 56 genes (revised to 59 genes in 2016) that are significantly associated with serious health conditions, for which we have treatment or prevention strategies, and for which we have sensitive and specific tests [29]. Various characteristics found in NGS make it a potentially powerful tool for NBS. A range of disorders can be analyzed with a single assay directly, samples can reduce costs, and can largely be automated. In addition to differentiation between borderline or ambiguous laboratory screening results, DNA sequencing can expand the analytical value of NBS results, especially if variable presentation is a key feature, such as in IEM [77].

Whole exome sequencing is routinely used and is gradually being optimized for the detection of rare and common genetic variants in humans [78,79,80,81,82]. While WES is increasingly being optimized for the detection of mutations in cumulative parts of the protein-coding regions, WGS is gradually becoming an attractive option. In contrast, for the time being, WGS is more costly compared to WES, but its cost is expected to reduce rapidly. However, the exome covers only 1% of the human genome: thus, any DNA variation in the non-protein-coding region will obviously be missed. Moreover, copy number and structural variations “CNVs and SVs”, as well as some insertions, deletions or block substitutions are difficult to detect in exome capture data [83]. Nevertheless, due to the reducing cost and wider coverage of WGS, it is gradually becoming attractive as an alternative method. Additionally, variants outside the protein-coding regions remain difficult to interpret. Therefore, public or private research and diagnostic laboratories tend initially to search for coding variants, which can mostly be detected by WES [46,84,85]. The incorporation of NGS in NBS will increase the number of uncertain variants, simultaneously increasing the burden on the parents and the care providers [86].

The performance of genomic sequencing technology and the capability of interpreting variants in asymptomatic newborns creates part of the barriers to utilize this technology in any screening attempt. Even more, how parental informed consent can be obtained, the type of information that should be offered, and how the possible results will impact families are critical issues [87].

A plausible alternative approach to “biochemical based” NBS would be applying NGS technologies to screen for mutations in the relevant disease predisposition genes. A key driving force for the “targeted NBS panels” approach is the rapidly decreasing cost of massively parallel sequencing coupled with the vastly improved read depths and accuracy of the sequencing platform [88].

Presently, nongenetic NBS programs do not need parental consent as they are primarily aimed for the newborn’s profit and are offered without any cost. Reporting information, which may impact the decisions of familial reproduction, also represents the secondary aim. On the other hand, NBS by genetic sequencing may require specific consent to be given by the parents, as all genetic testing does. One way to avoid these potential objections is to have mandatory NBS by WES/WGS for the disorders that could lead to immediate impact and benefit for the infant’s health, and request for consent for other diseases (*Tarini and Goldenberg, 2012*). Genetic counselling for the families is crucial to obtain consent for additional diseases to be investigated in the NBS [88,89]. To warrant the importance of mandatory screening for genetic disorders that is of direct benefit to the newborn, parental consent for screening is required where direct benefit criteria are not met.

Currently, too many queries have arisen on the application of WGS techniques in NBS with a high price, especially in dealing with the newborns admitted to ICU with dangerous diseases. Should we start with them? How can we prioritize the cases to be investigated and the criteria for this selection, and what are the standards of lab investigations [88]? The clinical sensitivity and specificity of WGS can be assessed through the implementation of NGS and careful evaluation of pilot studies in the targeted population for screening in the NBS [90].

Targeted sequencing rather than an exome- or genome-wide approach enables the addition of screening programs to continue on a disorder-by-disorder basis, as recommended by the UK National Screening Committee, rather than in response to any technological imperative [91,92,93]. NGS is increasingly being utilized in clinical diagnostics through targeted gene panels, WES and WGS, and can even be performed on DNA extracted from the DBSs [46,94].

Technological challenges with short-read NGS technologies remain significant in highly homologous genomic regions, such as pseudogenes or paralogous genes and need to be considered when implemented in screening programs [95]. There is a technological challenge with the paralogous genes or pseudogenes due to the failure of the short reads in mapping to the specific gene [96].

Large homologous regions are successfully covered by the new technology of third generation long-read sequencing paralogous SMN1 and SMN2 genes.

Many ethical implications have been demonstrated from the application of genome testing within the NBS program, whether the whole genome, only the coding region (exome) or for specific genes, due to boundaries of the bioinformatics about identified variants’ interpretation, uncertain probable management, psychological issues for the family, irrelevant therapy, increased medical cost, negative impact on family insurance and, lastly, the difficulty in consent obtaining from the families [43].

A scientifically-based recommendation has been issued from the Human Genome Organization (www.hugointernational.org/comm_hugoethicscommittee.php, accessed on 5 September 2021) stating that “the primary objective of genome sequencing in NBS should be the identification of gene variants conferring a high risk of preventable or treatable conditions, for which treatment has to start in the newborn period or in early childhood” [44,97].

## 13. Conclusions and Recommendations

The WGS and WES for clinical applications are now an integral part of medical genetics practice. The significant reduction in sequencing costs and availability have raised the inquiry about whether current practiced NBS technology should be shifted to WGS or even WES. Although the genome sequencing for newborns is beneficial for the detection of many disorders in its early stage, many ethical concerns may arise from its mandatory application as a routine in NBS.

For the implementation of a robust technology such as NGS in a mass NBS program, the main focus should not be just technologically biased; it should also be tested for its long- and short-term impact on the family and the child. The crucial question here is whether large-scale genomic sequencing can provide useful medical information beyond what current NBS is already providing and at what economical and emotional cost? Currently, the topic of newborn genome sequencing as a public health initiative remains argumentative. Thus, an answer has not yet been found to the question: NGS for newborn screening—are we there yet?

## Figures and Tables

**Table 1:** NBS Programs in some selected countries.

Country	Disorders	Others
**Saudi Arabia [15]**	ASA-βKT–BTD–CAH–CH–CIT-GA1–GALT–HMG–IVA–MCAD–MCC–MMA–MSUD–PA–PKU–VLCAD	Tyr-I–HCU–PCD
**USA**	ASA–BTD–βKT–CAH–CCHD–CF–CH–CIT–CTD–CUD–GA1–GALT-Hb S/b Th–HCLS–HCU–HMG–IVA–LCHAD–MCC-MMA^1^-MMA^2^–MSUD–PA–PKU-SC Disease–SCA–TFP-Tyr1–VLCAD	https://www.hrsa.gov/advisory-committees/heritable-disorders/rusp/index.html (accessed on 5 September 2021)
**UK**	CF–CH–GA1–HCU–IVA–MCAD–MSUD–PKU–SCA	https://www.gov.uk/government/collections/newborn-blood-spot-screening-programme-supporting-publications (accessed on 5 September 2021)
**China**	ARG1–CTD-GA1–HCU–HHH–HPA–IVA–MCAD–MCC–MMA–MSUD–PA–SBCAD–SCAD–TFP–Tyr–VLCAD	https://www.ncbi.nlm.nih.gov/pmc/articles/PMC3565663/ (accessed on 5 September 2021)
**Brazil**	Phase I: PKU and CH-Phase II: SCA and other hemoglobinopathies-Phase III: CF-Phase IV: CAH and BTD	https://www.researchgate.net/publication/6145921_Newborn_screening_A_national_public_health_programme_in_Brazil (accessed on 5 September 2021)
**Australia**	ARG1–ASA–βKT–CACT–cblC–CF–CH–CIT-CPT1-CPT2–CTD-GA1–GALT–HCLS–HCU–HMG–IBD–IVA–LCHAD–MADD–MCAD–MCC–MGH–MMA-MMA^1^–MSUD–PA–PKU-Pterin defect–SBCAD–SCAD–SCHADD–TFP-Tyr1-Tyrosine aminotransferase deficiency-VLCAD	https://www.racgp.org.au/getattachment/d717b2a9-c887-4510-805c-9378395a5c2f/attachment.aspx?disposition=inline (accessed on 5 September 2021)
**Qatar**	CH–CAH-PKU–HPA-BS–ASA–MSUD-HCY-CIT–Tyr II-GALT-BIOT-MMA–PROP-GA1-IVA–3MCC-MAD-IBDH-MCAD–VLAD-LCHAD-SCAD-CTD-CPT-I,II-HMG-βKT	https://www.nature.com/articles/gim200998/tables/2 (accessed on 5 September 2021)
**Uruguay**	BH_4_ deficiency–CAH–cblC–CF–CH–CIT–CUD–Hemoglobinopathies–HPA-Maternal B12 deficiency–MCAD–MCC–MMA^1^–PKU	https://doi.org/10.1590/2326-4594-JIEMS-2021-0008 (accessed on 5 September 2021)

Argininemia (also known as Arginase 1 deficiency, ARG1), argininosuccinic aciduria (ASA), tetrahydrobiopterin deficiency (BH4 deficiency), beta-ketothiolase deficiency (βKT), biotinidase deficiency (BTD), Carnitine-acylcarnitine translocase deficiency (CACT), congenital adrenal hyperplasia (CAH), Cobalamin C defect (cblC), critical congenital heart disease (CCHD), cystic fibrosis (CF), congenital hypothyroidism (CH), citrullinemia (CIT), Carnitine palmitoyltransferase type 1 deficiency (CPT1), Carnitine palmitoyltransferase type 2 deficiency (CPT2), Carnitine transport defect (CTD), Carnitine uptake defect (CUD), glutaric aciduria type 1 (GA1), galactosemia (GALT), hemoglobin S/beta thalassemia (Hb S/b Th), holocarboxylase synthetase deficiency (HCLS), homocystinuria (HCU), hyperornithinemia-hyperammonemia-homocitrullinuria syndrome (HHH), 3-hydroxy-3-methylglutaric aciduria (HMG), hyperphenylalaninemia (HPA), Isobutyryl-CoA dehydrogenase deficiency (IBD), isovaleric acidemia (IVA), long-chain 3-hydroxyacyl-CoA dehydrogenase deficiency (LCHAD), multiple acyl CoA dehydrogenase deficiency (MADD), medium-chain acyl-CoA dehydrogenase deficiency (MCAD), 3-methylcrotonyl-CoA carboxylase deficiency (MCC), 3-Methylglutaconyl-CoA hydratase deficiency (MGH), methylmalonic acidemia (MMA), MMA due to methylmalonyl-CoA mutase deficiency (MMA^1^), MMA due to cobalamin disorders (MMA^2^), maple syrup urine disease (MSUD), propionic acidemia (PA), primary carnitine deficiency (PCD), phenylketonuria (PKU), 2-methylbutyryl-CoA dehydrogenase deficiency (also known as short/branched chain acyl-CoA dehydrogenase (SBCAD) deficiency), hemoglobin SC disease (SC Disease), sickle cell anemia (SCA), short chain acyl-CoA dehydrogenase deficiency (SCAD), short chain hydroxyacyl-CoA dehydrogenase deficiency (SCHADD), trifunctional protein deficiency (TFP), tyrosinemia (Tyr), tyrosinemia type 1 (Tyr1), very long-chain acyl-CoA dehydrogenase deficiency (VLCAD).

**Table 2:** Consanguinity rates in Saudi Arabian populations. (Tadmouri G.O., Nair P., Obeid T., Al Ali M.T., Al Khaja N., et al. (2009) Consanguinity and reproductive health among Arabs. Reprod Health 6: 17).

Location	Collection Period *	Sample Size	>1C,1C *	Overall *	Reference
**Riyadh**	1983–1986	4497	31	54.3 [?]	[43]
**Riyadh**	1993	2001	28.4	51.1 [1C, 1.5C, 2C,<2C]	[44]
	1995 (?)	3212	25.8	56.8 [1C, 1.5C, 2C,<2C]	[45]
**Dammam**	1998 (?)	1307	39.3	52 [1C,1.5C, 2C,<2C]	[46]
**Al-Baha**	2004–2005	487	29	42.1 [?]	[45]
**Al-Jawf**	2004–2005	593	34.8	53.5 [?]	[45]
**Assir**	2004–2005	833	24.6	44.5 [?]	[45]
**Eastern Province**	2004–2005	1032	33.3	57.8 [?]	[45]
**Gizan**	2004–2005	565	33	53.5 [?]	[45]
**Hail**	2004–2005	505	25.1	48.9 [?]	[45]
**Madinah**	2004–2005	618	39.2	67.2 [?]	[45]
**Makkah**	2004–2005	2278	32.4	55.9 [?]	[45]
**Najran**	2004–2005	472	28.4	66.7 [?]	[45]
**Northern Borders**	2004–2005	504	31.4	63.9 [?]	[45]
**Qassim**	2004–2005	713	29.6	46.7 [?]	[45]
**Riyadh**	2004–2005	2522	42.3	60 [?]	[45]
**Tabuk**	2004–2005	432	28.3	60 [?]	[45]
**All Saudi Arabia**	2004–2005	11,554	33.6	56 [?]	[45]

* (Quoted with permission from the author of the article titled: Towards a Uniform Newborn Screening Panel in the Kingdom of Saudi Arabia. Dr. Ahmed Bashir et al., Jubail Hospital). Abbreviations: [?] = Unknown year of sampling or unknown types of consanguineous marriages; [>1C] = Double first-cousin marriage; [1C] = First-cousin marriage; [<1C] = Marriage beyond first-cousins; [1.5C] = First-cousin once removed marriage; [2C] = Second-cousin marriage; [<2C] = Marriage between distant relatives beyond second-cousins [47].

**Table 3:** Molecular technology applications in Wisconsin routine newborn screening.

Molecular Application	Example Condition	Molecular Marker	Technology
**First-tier markers**	Severe combined immunodeficiency	T-cell receptor excision circles	Real-time polymerase chain reaction (PCR)
	Spinal muscular atrophy	Homozygous *SMN1* exon 7 deletion	Real-time PCR
**Second-tier markers**	Cystic fibrosis	*CFTR* variants	Next generation sequencing
Supplemental “just-in-time” information *	Galactosemia	*GALT* c.563A>G, c.404C>T, and c.940A>G	Tetra-primer amplification refractory mutation system (Tetra-primer ARMS)–PCR
	Maple syrup urine disease	*BCKDHA* c.1325 T>A	Tetra-primer ARMS–PCR
	Sickle cell disease	*HBB* c.20 A>T	Sanger sequencing
	Pompe disease	*GAA* coding region and intron-exon junctions	Sanger sequencing
	Spinal muscular atrophy	*SMN2* copy number	Droplet digital PCR

* The determination of screening positive is dependent on the first-tier result, and the information of gene variant “just-in-time” is aimed to assist physicians for better interpretation of the screening results and be better prepared for their initial communication and discussion with families regarding NBS positive results (https://www.ncbi.nlm.nih.gov/pmc/articles/PMC7712315/ accessed on 5 September 2021).

**Table 4:** Some novel, common and founder mutations in Saudi population.

#	Disorder	Gene	Nucleic Acid Change	Effect
1	ASA	ASL	c.1060C>T	p.Q354X
			c.556C>T	p.R186W
			c.343G>T	p.D115Y
			c.469G>A	p.G157R
			c.496C>A	p.P166S
			c.544C>T	p.R182X
			c.1081G>T	p.G361X
			IVS13+5G>C	
2	BD	BTD	c.654G>C	p.E218D
			c.466C>T	p.Q156X
			del490A-491G	Frame shift
			del544A	Frame shift
			Inser or del G76:d7i3	
			c.38G>T	p.C33F
3	CAH	CYP21A2	c.952 C>T	p.Q318X
			c.290-13 C>G	IVSK13C>G
			Exon 6&8 del	
		CYP11B1	c.780 G>A	p.W260X
4	CIT	ASS1	c.1087 C>A	p.R363W
5	GA-I	GCDH	c.1208A>G	p.H403R
			c.1169G>C	p.G390A
			c.1144G>A	p.A382T
			c.1060G>C	p.G354R
			c.937C>T	p.R313W
6	HMG	HMGCL	c.122G>A	p.R41Q
			F305 (shift K2)	
7	MCAD	ACADM	c.262C>T	p.T121I
			c.347G>A	p.C116Y
		MCCC2		
8	MMA	MUT	c.329A>G	p.Y110C
			c.2200C>T	p.Q734X
9	PA	PCCA	c.350G>A	p.G117D
			c.425G>A	p.G142D
10	PKU	PAH		p.R261
11	VLCAD	ACADVL	IVS16+6GC del	
			c.65C>A	p.S22X
12	*Classic Homocystinuria*	CBS	c.969G>A	p.W323X
13	*Familial chloride diarrhea*	SLC26A3	c.559G>T	p.G187X
14	*GA-II*	ETFDH	c.786G>T	p.L262F
15	*Glycogen storage disease type III GSDIII*	AGL	IVS32K12AA>G	
16	*Lipoid congenital adrenal hyperplasia*	STAR	c.545G>A	p.R182H
17	*M* *aroteaux-Lamy Syndrome*	ARSB	c.753C>G	p.Y251X
18	*Niemann-Pick Type B*	SMPD1	c.1267C>T	p.H423Y
			c.1734G>C	p.K578N
19	*Osteoporosis*	CA2	c.232+1G>A	
20	*Papillon-Lefevre Syndrome*	CTSC	c.815G>C	p.R272P
21	*Sanjad-Sakati syndrome*	TBCE	155_166del	
22	*Wilson disease*	ATP7B	c.2230T>C	p.S744P
			c.4196A>G	p.Q1399R
			4193delC	
23	*Wouldhouse-Sakati syndrome*	DCAF17	436delC	

**Table 5:** Primers for amplifying and sequencing of some targeted mutations (gene panel sequencing) listed in Table 4.

#	Gene	Mutation	Primer Label	Sequence
**1**	ASL	D115Y	ASL_F1	CCTCTGGGGGTATAGACCGT
			ASL_R1	AAGGTTGGGACAACACGGAG
		G157R P166S R182X R186W	ASL_F2	TCCACCCGAGCTTCTGCT
			ASL_R2	CAGCTCTGTCAATCCCTAAGGCT
		IVS13+5G>C Q354X	ASL_F3	GCTCCTGATGACCCTCAA
			ASL_R3	GAGCGAGCACACCTCTCC
		G361X	ASL_F4	CAGAGCCGAGTGGGTAAGAG
			ASL_R4	TTTGCGGACCAGGTAATAGG
**2**	ACADM	C116Y	ACADM_F1	CTGTAGGAGGTCTTGGACTTGG
		T121I	ACADM_R1	GCCTCGAAATCAGAACTCCA
**3**	CBS	W323X	CBS_F1	GGGTCCTACCGCCTAGACAC
			CBS_R1	GTCGGTGGCTGACTGAGG
**4**	AGL	IVS32-2A>G	AGL_F1	GCAGTGATATGGTTTACTGTGG
			AGL_R1	GTCTTTGCAGTAGTCTCCGGG
**5**	HMGCL	R41Q	HMGCL_F1	TGGGCACTTTACCAAAGCGG
			HMGCL_R1	TGTCAACTGCCATTGCACCTA
		F305 (shift-2)	HMGCL_F2	GGCATACCATGACTTACCGCA
			HMGCL_R2	TGAGCCACTTTGGAGCTAGT
**6**	ATP7B	S744P	ATP7B_F1	CTAGAACCTGACCCGGTGAC
			ATP7B_R1	CTCATGTGACCTGACAGCTGCT
		4193delC p.Q1399R	ATP7B_F2	AGAGGCCTTCACCAGGC
			ATP7B_R2	GCTGACCTGGTCCCATGGTG
**7**	SLC26A3	G187X	SLC26A3-F1	CTGAGTATGATGGTGGGACTAGC
			SLC26A3-R1	CAGTCAAGATGAACAGATTGAGGTG
**8**	CA2	c.232+1G>A	CA2_F1	CAGCCAAGTATGACCCTTCC
			CA2_R1	GCTCGGAAAACAGCTACTGG
**9**	STAR	R182H	STAR_F1	GGCTGAGTCGTGATTCTGGT
			STAR_R1	CTGATGACACCCTTCTGCTCAG
**10**	DCAF17	c.436delC	DCAF17_F1	CACTGACTGCTCATAATTGGCT
			DCAF17_R1	ATTTCATGGGCCACAGGTTTC
**11**	TBCE	c.155_166del12bp	TBCE_F1	CAATCCCGAGAGAGGAAAGC
			TBCE_R1	CTTGACTAAATGACCGTGCTGAT

## Data Availability

Not applicable.
